# COMMENTARY ON “EFFECTS OF INCENTIVE SPIROMETER ADDED TO STANDARD REHABILITATION IN PREVIOUSLY HOSPITALIZED ADULTS WITH POST-COVID-19 CONDITION: A RANDOMIZED CONTROLLED TRIAL”

**DOI:** 10.2340/jrm.v58.46243

**Published:** 2026-07-09

**Authors:** Etika RANA

**Affiliations:** Maharishi Markandeshwar Institute of Physiotherapy and Rehabilitation (MMIPR), Maharishi Markandeshwar (Deemed to be University), Mullana, Haryana, India. E-mail: etikarana777@gmail.com

*To the Editor*,

The recently published article, “Effects of Incentive Spirometer Added to Standard Rehabilitation in Previously Hospitalized Adults With Post-COVID-19 Condition: A Randomized Controlled Trial” by Kloni et al. (1) offers valuable insight into rehabilitation strategies for individuals experiencing persistent respiratory impairment following coronavirus disease. The authors should be commended for employing a randomized design to evaluate an accessible intervention in a clinically important yet evolving field of post-COVID-19 rehabilitation.

While the study contributes meaningful preliminary evidence, several methodological and analytical considerations may further strengthen interpretation of the findings.

First, although multiple clinically relevant outcomes, including pulmonary function, dyspnoea, mobility, endurance, quality of life, muscle strength, and functional independence – were assessed, the sample size estimation appears to have been derived from a single respiratory endpoint. In randomized studies evaluating numerous outcomes, explicit pre-specification of outcome hierarchy and clarification regarding multiplicity handling are important to reduce the possibility of inflated type I error and selective emphasis on statistically significant findings (2, 3). Future investigations may benefit from clearly distinguishing confirmatory from exploratory outcomes and reporting multiplicity-adjusted inference where appropriate.

Second, additional transparency regarding the analytical strategy may improve reproducibility and interpretation. The authors report baseline-adjusted comparisons between groups; however, further clarification regarding model specification, assumptions, and adjusted effect estimates would enhance methodological transparency. Current recommendations for randomized trials support covariate-adjusted analyses, particularly analysis of covariance (ANCOVA), because such approaches may improve precision and reduce residual baseline imbalance (4, 5). More detailed reporting of these procedures could strengthen confidence in the robustness of treatment estimates.

Third, the choice of pulmonary outcome measurement warrants consideration. Incentive spirometry primarily aims to promote inspiratory effort and lung expansion, whereas peak expiratory flow (PEF), the principal respiratory endpoint, predominantly reflects expiratory airflow. Although improvement in PEF may indicate respiratory benefit, broader pulmonary function measures including indices reflecting lung volume and ventilatory performance could potentially offer a more comprehensive assessment of physiological recovery and intervention-specific mechanisms in individuals with post-COVID-19 condition (6, 7).

Additionally, while statistically significant improvements were observed for selected outcomes, contextualizing these findings through clinically meaningful thresholds or effect-size interpretation may improve understanding of their practical relevance to patient recovery. Statistical significance alone may not always translate into meaningful functional improvement in rehabilitation settings (8).

These observations are intended in a constructive spirit and should not detract from the contribution of this study. Rather, the work by Kloni et al. provides an important foundation for future investigations seeking to refine respiratory rehabilitation approaches for individuals with post-COVID-19 condition through transparent analytical reporting and comprehensive physiological assessment.
