# Supplementary material for: COMMENTARY ON “EFFECTS OF INCENTIVE SPIROMETER ADDED TO STANDARD REHABILITATION IN PREVIOUSLY HOSPITALIZED ADULTS WITH POST-COVID-19 CONDITION: A RANDOMIZED CONTROLLED TRIAL”
Source: J Rehabil Med. 2026 Jul 9;58:46243. doi: 10.2340/jrm.v58.46243 (PMC13397338; doi:10.2340/jrm.v58.46243)
Supplement: Related texts

# RESPONSE TO COMMENTARY ON “EFFECTS OF INCENTIVE SPIROMETER ADDED TO STANDARD REHABILITATION IN PREVIOUSLY HOSPITALIZED ADULTS WITH POST-COVID-19 CONDITION: A RANDOMIZED CONTROLLED TRIAL”

**Authors:** Marina KLONI, Alexandros HERACLIDES, Theognosia PANTELI, Alexios KLONIS, Panagiotis RENTZIAS, Christos KARAGIANNIS

**Affiliations:** 1 Physiotherapy Department, Rehabilitation Centre EDEN, Larnaca; 2 Department of Health Sciences, European University of Cyprus, Nicosia, Cyprus; 3 Department of Anaesthesiology, The Newcastle Upon Tyne Hospitals NHS Foundation Trust, Newcastle, UK

*To the Editor*,

We thank the author for their interest in our study and for their constructive comments.

Regarding sample size estimation, we acknowledge that the calculation was based on peak expiratory flow (PEF), which served as the primary respiratory endpoint. At the time the study was designed, evidence regarding the effects of incentive spirometry in individuals with post-COVID-19 condition was limited, and the available literature provided the basis for estimating the expected treatment effect (9). While several additional outcomes were assessed to capture the multidimensional nature of post-COVID-19 condition, multiplicity was addressed through application of the Benjamini–Hochberg false discovery rate correction, as reported in the Methods section (2).

We fully agree that baseline-adjusted analyses, such as ANCOVA, represent the gold standard for parallel-group randomized trials due to their superior ability to optimize statistical precision and mitigate residual baseline imbalances (4, 10). In our study, a change-score approach (Yfollow-up – Ybaseline) was utilized to assess the intervention effect. We acknowledge that this simpler approach generally exhibits lower statistical power than an ANCOVA model. However, because our randomization process was highly successful, resulting in well-balanced baseline characteristics and outcome measures between the 2 groups, the risk of bias due to regression to the mean or baseline confounding is minimized. While we recognize that an ANCOVA model would have provided narrower confidence intervals, we are confident that our simpler, transparent change-score analysis offers a conservative and valid estimate of the treatment effect (4, 10).

We appreciate the authors’ comments regarding the choice of pulmonary outcome measures. Although incentive spirometry primarily targets inspiratory effort and lung expansion, PEF was selected because it is a practical, inexpensive, and clinically accessible measure that can be readily implemented in rehabilitation settings (11). We agree that future investigations incorporating additional measures of pulmonary function, including inspiratory parameters and lung volume assessments, may further elucidate the physiological mechanisms underlying recovery.

Finally, we agree that clinical relevance is an important consideration when interpreting rehabilitation outcomes. Accordingly, in the Discussion we contextualized the observed improvements using available evidence regarding clinically meaningful changes in both PEF and MRC dyspnoea scores (12, 13). The significant between-group differences observed in these outcomes were interpreted not only statistically but also in terms of their potential functional relevance for individuals with post-COVID-19 condition.

We thank the author for their thoughtful observations and welcome continued discussion aimed at strengthening the methodological and clinical evidence base for post-COVID-19 rehabilitation research.

## References

[1] Kloni M, Heraclides A, Panteli T, Klonis A, Rentzias P, Karagiannis C. Effects of incentive spirometer added to standard rehabilitation in previously hospitalized adults with post-COVID-19 condition: a randomized controlled trial. J Rehabil Med 2026; 58: jrm44926. 10.2340/jrm.v58.4492642084202 PMC13154367

[2] Benjamini Y, Hochberg Y. Controlling the false discovery rate: a practical and powerful approach to multiple testing. J R Stat Soc Series B 1995; 57: 289–300. 10.1111/j.2517-6161.1995.tb02031.x

[3] Ioannidis JPA. Why most discovered true associations are inflated. Epidemiology 2008; 19: 640–648. 10.1097/EDE.0b013e31818131e718633328

[4] Vickers AJ, Altman DG. Analysing controlled trials with baseline and follow up measurements. BMJ 2001; 323: 1123–1124. 10.1136/bmj.323.7321.112311701584 PMC1121605

[5] Schulz KF, Altman DG, Moher D; CONSORT Group. CONSORT 2010 statement: updated guidelines for reporting parallel group randomised trials. BMJ 2010; 340: c332. 10.1136/bmj.c33220332509 PMC2844940

[6] Spruit MA, Holland AE, Singh SJ, Tonia T, Wilson KC, Troosters T. COVID-19: interim guidance on rehabilitation in the hospital and post-hospital phase from a European Respiratory Society and American Thoracic Society-coordinated international task force. Eur Respir J 2020; 56: 2002197. 10.1183/13993003.02197-202032817258 PMC7427118

[7] Hyatt RE, Scanlon PD, Nakamura M. Interpretation of pulmonary function tests: a practical guide. 4th ed. Philadelphia, PA: Wolters Kluwer; 2014.

[8] Sullivan GM, Feinn R. Using effect size – or why the P value is not enough. J Grad Med Educ 2012; 4: 279–282. 10.4300/JGME-D-12-00156.123997866 PMC3444174

[9] Soril LJJ, Damant RW, Lam GY, Smith MP, Weatherald J, Bourbeau J, et al. The effectiveness of pulmonary rehabilitation for post-COVID symptoms: a rapid review of the literature. Respir Med 2022; 195: 106782. 10.1016/j.rmed.2022.10678235272262 PMC8887973

[10] Egbewale BE, Lewis M, Sim J. Bias, precision and statistical power in analysis of covariance compared with change score methods in randomized controlled trials: a simulation study. BMC Med Res Methodol 2014; 14: 49. 10.1186/1471-2288-14-4924712304 PMC3986434

[11] Walker PP, Key AL. How to perform peak flow and spirometry tests. BMJ 2016; 353: h6159. 10.1136/sbmj.h615931055386

[12] Spruit MA, Singh SJ, Garvey C, ZuWallack R, Nici L, Rochester CL, et al. An official American Thoracic Society/European Respiratory Society statement: Key concepts and advances in pulmonary rehabilitation. Am J Respir Crit Care Med 2013; 188: e13–e64. 10.1164/rccm.201309-1634ST24127811

[13] Bestall JC, Paul EA, Garrod R, Garnham R, Jones PW, Wedzicha JA. Usefulness of the Medical Research Council (MRC) dyspnoea scale as a measure of disability in patients with chronic obstructive pulmonary disease. Thorax 1999; 54: 581–586. 10.1136/thx.54.7.58110377201 PMC1745516
